# Development of diagnostic and point of care assays for a gammaherpesvirus infecting koalas

**DOI:** 10.1371/journal.pone.0286407

**Published:** 2023-06-01

**Authors:** Belinda R. Wright, Martina Jelocnik, Andrea Casteriano, Yasmine S. S. Muir, Alistair R. Legione, Paola K. Vaz, Joanne M. Devlin, Damien P. Higgins

**Affiliations:** 1 Koala Health Hub, Sydney School of Veterinary Science, University of Sydney, Camperdown, New South Wales, Australia; 2 Centre for Bioinnovation, University of The Sunshine Coast, Sippy Downs, Queensland, Australia; 3 Asia Pacific Centre for Animal Health, Melbourne Veterinary School, Faculty of Science, University of Melbourne, Parkville, Victoria, Australia; Nagasaki University, JAPAN

## Abstract

The recent listing of koala populations as endangered across much of their range has highlighted the need for better management interventions. Disease is a key threat to koala populations but currently there is no information across the threatened populations on the distribution or impact of a gammaherpesvirus, phascolarctid gammaherpesvirus 1 (PhaHV-1). PhaHV-1 is known to infect koalas in southern populations which are, at present, not threatened. Current testing for PhaHV-1 involves lengthy laboratory techniques that do not permit quantification of viral load. In order to better understand distribution, prevalence and impacts of PhaHV-1 infections across koala populations, diagnostic and rapid point of care tests are required. We have developed two novel assays, a qPCR assay and an isothermal assay, that will enable researchers, clinicians and wildlife managers to reliably and rapidly test for PhaHV-1 in koalas. The ability to rapidly diagnose and quantify viral load will aid quarantine practices, inform translocation management and guide research into the clinical significance and impacts of PhaHV-1 infection in koalas.

## Introduction

Koala populations across the Australian states of New South Wales (NSW), Queensland (Qld) and the Australian Capital Territory (ACT) have recently been listed as endangered [1], with disease being a key threat [2, 3]. *Chlamydia pecorum* infections are highly prevalent across koala populations [4], with ocular and urogenital infections potentially leading to blindness, urinary tract disease, infertility or host death [5]. Koala retrovirus (KoRV) is another pathogen infecting koalas that has been associated with chlamydial infections and neoplasia [6, 7]. Two recently discovered gammaherpesviruses, phascolarctid gammaherpesvirus 1 (PhaHV-1) [8] and phascolarctid gammaherpesvirus 2 (PhaHV-2) [9], infect koalas, with research on distribution and prevalence currently limited to South Australian and Victorian koala populations [10, 11]. Gammaherpesviruses are one of three subfamilies of double-stranded DNA viruses, *Herpesviridae*, along with the *Alpha*- and *Betaherpesvirinae*. Members of the *Herpesviridae* are well known for establishing latency, with the site of their latent infections being one of the distinguishing features of the three subfamilies [12]. In many host species gammaherpesviruses establish latent infections in B and T lymphocytes with active infections occurring in epithelial cells [13, 14]. Stress and immunosuppression may activate latent gammaherpesvirus infections and this can result in lymphoproliferative disorders in immunocompromised individuals [15, 16]. While the latent site of PhaHV infection has not been confirmed, it has been detected in koala liver, spleen, bone marrow and lymph nodes [17], suggesting lymphocytes as a likely candidate.

While the pathogenesis of the PhaHVs is not yet understood, herpesviruses have been identified as a potential risk to koala health and welfare [3]. This is in part due to the impact of co-infections which may be significant as some co-infections can impact disease progression. For example, research using mouse models has demonstrated that acute infection with a gammaherpesvirus can inhibit anti-malarial immune responses potentially leading to death [18]. In human studies latent infection by both alpha- and beta-herpesviruses can be stimulated to replicate or to infect normally non-susceptible cells by *Chlamydia trachomatis* infection, and was also able to induce persistence of *C*. *trachomatis in vitro* [19–21]. One human case study has also found a potential association between co-infection with a gammaherpesvirus and *C*. *pneumoniae*, and the development of cutaneous lymphoma [22]. In koalas, significant associations have been identified between PhaHVs and detection of *C*. *pecorum* [10, 23], as well as between PhaHV-1 infection and presence of koala retrovirus (KoRV) [10]. These interactions suggest that immune status and co-infections may be playing a role in disease progression in koalas. For precautionary risk management in koala rehabilitation facilities, PhaHV-1 is considered of higher priority due to its association with KoRV, and its association with older koalas suggesting it is horizontally transmitted, while PhaHV-2 may be acquired early in life through mother to offspring transmission [10, 11].

Understanding and mitigating any role of gammaherpesvirus infection in koala health requires new sensitive and specific diagnostic tests that can detect active infection and quantify viral load, as well as provide rapid results to inform segregation of animals in rehabilitation facilities. In South Australian populations, PhaHVs were detected in 72% of koalas, but detection of the virus from mucosal sites [mostly likely representing active rather than latent infection] was only detected in 54% of koalas [11]. PhaHV infection is typically determined using urogenital swabs [10, 11], possibly because these are routinely collected for chlamydial testing, though the virus is also detectable in oropharyngeal and ocular swabs [10]. Currently, diagnostic testing for PhaHVs involves a nested PCR of the *dpol* gene encoding the viral DNA polymerase catalytic subunit followed by high-resolution melt (HRM) curve analysis [10], which is a laborious process and not suited to quantification, rapid diagnostics or in-clinic settings. Quantitative PCR (qPCR) is considered the gold standard diagnostic assay due to sensitivity and specificity and provides real-time pathogen detection as well as an estimate of viral load [24]. Loop-mediated isothermal amplification (LAMP) is a low-cost rapid pathogen detection method [25] that can be run on a portable instrument, making LAMP highly suitable for both field and in-clinic point of care testing, where immediate decisions are required to limit potential transmission. The utility of chlamydial rapid isothermal testing, including rapid sample processing, has been demonstrated in koala clinical practice to detect *C*. *pecorum* infections [26, 27].

This study aimed to develop and validate a qPCR assay for use in research settings to detect and quantify load of PhaHV-1 infection. Additionally, we developed and evaluated a LAMP assay as a rapid diagnostic tool for use in point of care testing in clinical and field settings. These assays have complimentary utility across different settings, allowing research laboratories and veterinary clinics to work collaboratively in gaining vital information on distribution, prevalence and impacts of PhaHV infection.

## Methods

### Samples used for qPCR development and evaluation

Three known positive PhaHV-1 DNA samples extracted from koala swabs from a previous study [10], were used in qPCR assay development and validation. Additional DNA samples were obtained from freshly extracted koala swab samples kept at the Koala Health Hub (University of Sydney). Swab samples were taken from multiple anatomical sites to determine the best sample type for PhaHV-1 detection using qPCR. Sampling of koalas was conducted under the University of Sydney Animal Ethics Approval Number 2021/1975, NSW NPWS Scientific License SL102379 and Qld NPWS WA0019256. We tested a total of 100 swab samples taken on the same date from 47 koalas. Paired oropharyngeal (n = 47) and urogenital (n = 47) swabs were taken from the same koalas as well as ocular (n = 6) swabs from a subset of the same koalas. DNA from swabs was extracted using the Kingfisher MagMAX™ CORE Nucleic Acid Extraction Kit (ThermoFisher Scientific, Australia). Additionally, scat samples were obtained from a subset of 29 of the same koalas on the same date as swab collection.

### DNA extraction from scats

Single koala scat pellets were collected in individual 5 mL tubes containing 3 mL of filter-sterilised lysis buffer (100 mM Tris-HCl; 50 mM EDTA; 1% w/v SDS; pH8) and frozen at -20°C until DNA was extracted. DNA extraction was carried out using ISOLATE II Fecal DNA Kit (Meridian Bioscience®, Australia) according to the manufacturer’s instructions except for the following modifications: Lysis buffer (700 μL) and approximately 200 mg of scat from the collection tube were added to a Bashing Bead Lysis Tube and homogenized at 6 m/s for 30 s using a FastPrep®-24 instrument. Prior to samples being loaded onto a Spin II A Filter, an added centrifugation step (10 000 x g, 3 min) was carried out to pellet colloidal material using a 1.5 mL micro-centrifuge tube. DNA was eluted to a final volume of 100 μL in RNase-free water.

### Primer design

The *dpol* genes of both PhaHV-1 and PhaHV-2 (GenBank accession numbers: JN585829.1, JQ996387.1 respectively) were investigated for appropriate qPCR priming sites. Primers were designed using primer3plus [28], checked for dimerization with Oligoanalyzer [29], and specificity using NCBI BLAST [30]. Multiple primer sets were tested for PhaHV-1 and the primer set with the best amplification was chosen with a resulting product size of 103 bp (Table 1). The sequence available for PhaHV-2 had no appropriate priming sites due to GC content and repetitive bases in the target region as confirmed by testing multiple primer sets for PhaHV-2 *in silico* and in laboratory tests.

**Table 1:** qPCR primers for PhaHV-1 developed in this study.

*Primer*	*Sequence 5’-3’*	*Length*	*Tm*	*GC %*
PhaHV-1_fwd	GGGAAGAACTATGTTGGAACGC	22 bp	59.6 C	50.0
PhaHV-1_rev	TGAGTCCTTTTCGCTTGGGA	20 bp	59.2 C	50.0

### Sensitivity and specificity of novel qPCR assay

Quantitative PCR was conducted in 20 μL reactions which included forward and reverse primers at a final concentration of 250 nM, 1x SsoAdvanced Universal SYBR Green Supermix (Bio-Rad, Australia) and 2 μL DNA template. PCR conditions were an initial denaturation of 98°C for 3 min, followed by 40 cycles of denaturation at 98°C for 10 s and annealing/extension at 56°C for 20 s, and a final melt curve of 56–95°C at 0.5°C increments. A synthetic positive control was constructed using a pMG-Amp vector (Macrogen, South Korea) containing the target region and flanking sequence (166 bp) to generate a standard curve and calculate the limit of detection (LOD). The efficiency and LOD were calculated using a minimum of four replicates of 1 x 10^8^ to 1 x 10^4^ copies/μL, and ten replicates of 1 x 10^4^, 1 x 10^3^, 50, 25, 10, 5 and 1 copy/μL (S1 Table in S1 File).

Upon establishment of LOD and standard curve of the qPCR assay, we tested samples in duplicate using the reaction conditions as outlined above. Test qPCRs were conducted alongside a standard curve comprising the synthetic positive control at 10-fold dilutions (1 x 10^7^ to 1 copy/ μL) for quantification and 2 μL of PCR grade water as a no template control. Previously published primers amplifying β-actin [31] were used as a quality check for DNA quantity and quality and presence of inhibitors. A sample was considered positive if both duplicates at single or multiple anatomical swab sites successfully amplified β-actin and achieved a cycle threshold (Ct) of < 37 (equivalent to 12 copies, see Results) for PhaHV-1 and a melt at 81–81.5°C. Any sample with discordant results between duplicates was retested and samples that failed to amplify β-actin were re-run at 1:10 dilution.

To confirm our qPCR specificity, we tested DNA extracts from KoRV, *C*. *pecorum*, Macropodid herpesvirus 1 (MaHV-1) and PhaHV-2 positive samples. In addition, a subset of samples (N = 12) that produced a positive PhaHV-1 result (according to criteria above), together with amplicons from the three known positive PhaHV-1 DNA samples and PCR product amplified from the PhaHV-1 synthetic positive control were sent to Macrogen, South Korea for by Sanger bidirectional sequencing. Resulting forward and reverse sequences were used to make a consensus sequence for each sample and then subjected to BLASTn using the NCBI database [30] to check amplicon sequence identity.

### PhaHV-1 and -2 point-of-care test (LAMP) development and evaluation

In order to design LAMP primers for PhaHV-1 and PhaHV-2 *dpol* gene, three different LAMP primer design software were used: licensed LAMP Designer 1.15 software (Premier Biosoft, CA, USA), open-source Primer Explorer v5 (Fujitsu Limited, Japan) and whole Genome based LAMP primer designer (GLAPD) [32]. As for the qPCR assay, no appropriate primers were predicted for PhaHV-2 so further work was conducted for PhaHV-1 *dpol* only. Primers were evaluated for sequence specificity *in silico* using PrimerBLAST [30] and primer characteristics using online tools OligoEvaluator (http://www.oligoevaluator.com) and OligoAnalyzer [29]. Upon preliminary testing of primers using a Genie III fluorometer (to evaluate time to amplify, amplification and melt curves for each set using DNA extracted from PhaHV-1 culture), we selected the most optimal primer set for PhaHV-1, designed by GLAPD (Table 2). The outer F3/B3 primers amplify a 216 bp fragment of the PhaHV-1 *dpol* gene, near the 5’ end and distinct from the region targeted by our qPCR primers (S1 Fig in S1 File).

**Table 2:** LAMP primers for PhaHV-1 developed in this study.

*Primer*	*Sequence 5’-3’*	*Length*
F3	ACTTTGTTCCAGGACGTCTC	20 bp
B3	GTTTCCTTGGATGGGCTCC	20 bp
FIP	GCTGCCCCAGATGTTCTGAAGCCCCAAACACCCTACCCAATC	42 bp
BIP	TCTTCCCCAGTGACCATGGTCCAGCTCCTTCCCCCAGAATTA	42 bp
LF	AGTTAGGCACCAATCGCGTGAT	22 bp
LB	ATGGAAGACAAATTTACACCC	21 bp
F2	CCCAAACACCCTACCCAATC	20 bp
F1c	GCTGCCCCAGATGTTCTGAAGC	22 bp
B2	AGCTCCTTCCCCCAGAATTA	20 bp
B1c	TCTTCCCCAGTGACCATGGTCC	22 bp

### PhaHV-1 isothermal assay evaluation

All LAMP assays were performed in 25 μL reaction volumes, consisting of 15 μL Isothermal Master Mix ISO001 (Optigene, UK), 5 μL primer mix (at 0.2 μM F3 and B3, 0.8 μM FIP and BIP, and 0.4 μM LF and LB as per primer mix for regular reactions), and 1 μL template (for sensitivity assays only) or 5 μL template (for specificity and sample testing assays); run at 65°C for 30 min followed by denaturation step of 98–80°C at a rate of 0.05°C/s in the Genie III real-time fluorometer (Optigene, UK) to create a high-resolution melt curve. Positive (PhaHV-1 DNA) and negative (MilliQ water) controls were included in each assay.

To evaluate analytical sensitivity (LOD) of the PhaHV-1 isothermal assay, we used 1 μL of quantified PhaHV-1 DNA (as determined by qPCR of PhaHV-1 DNA extracted from a culture against the standard curve generated from serially diluted synthetic positive control described above) as a template in serial dilutions from 500 copies/reaction (tested in triplicate), and 100, 50, 10, 1 and 0 copies/reactions (all tested as five replicates). Each serial dilution set was prepared separately from the same quantified PhaHV-1 DNA, with dilutions tested in separate runs by the same operator (S2 Table in S1 File). The specificity of the newly developed PhaHV-1 LAMP assay was evaluated against a range of previously screened and confirmed DNA extracts from KoRV, *C*. *pecorum*, MaHV-1 and PhaHV-2 positive samples (S3 Table in S1 File).

### Isothermal testing of DNA and rapidly processed swab samples

The newly developed PhaHV-1 LAMP assay was evaluated against 70 DNA samples, previously extracted from ocular, nasal, urogenital and rectal mucosal swabs from 38 koalas, including asymptomatic individuals and with those signs of chlamydial and/or other clinical disease, available in the University of the Sunshine Coast sample collection. To evaluate the use of rapidly processed swabs in the PhaHV-1 assay, we used a total of 36 mucosal swabs (Minitip Rayon dry Aluminium shaft (Copan, Brescia, Italy) obtained from 16 ocular, 15 urogenital, one nasal and four rectal sites from 17 koalas. Swabs were processed in 300 μL of water by vortexing for 45 sec and heat lysis at 90°C for 10 min, as previously described [26, 33]. All swabs were collected by qualified veterinarians as a part of routine health and diagnostic investigations. The testing and use of these swabs were approved by University of the Sunshine Coast Animal Ethics approval exemption (ANE2057).

All LAMP reactions were run as described above, with 5 μL of the DNA samples or swab suspensions used as template in a reaction. In these assays, a swab suspension or DNA sample was considered negative if amplification time was > 27.45 min with no or below threshold melt, as this was considered below detection level. Inhibition of amplification in swab suspensions was also assessed with ‘spiking’ experiments, wherein we added 1000 copies of PhaHV-1 DNA/reaction to 14 aliquots (50 μL) of selected swab suspensions that tested negative. The remaining volume of the swab suspension was used for DNA extraction using QiaAMP DNA mini kit, as per manufacturer instructions (Qiagen, Australia). Similarly, to assess whether the extracted swab DNA may carry over inhibitors, we then spiked 50 μL of the DNA with 1000 copies of PhaHV-1 DNA per aliquot. Sample quality was evaluated using koala β-actin qPCR [31].

In order to evaluate LAMP relative to the newly developed PhaHV-1 qPCR assay, all 106 DNA samples consisting of 70 previously extracted and 36 freshly extracted DNA samples from mucosal swabs (separate to the sample set used in qPCR assay development) were also tested in duplicate with the PhaHV-1 qPCR assay as described above. Samples with a Ct < 37 or without a melt curve consistent with the positive controls, were considered negative.

### Statistical analyses

The performance of the tests conducted on the same samples was evaluated using EpiTools online epidemiological calculators using diagnostic test evaluation modules [34] with calculated Kappa and positive and negative agreement proportions, as well as McNemar’s Chi-squared value with 95% confidence. Agreement analysis was considered more appropriate than relative sensitivity and specificity, as both tests were novel and, therefore, neither could be designated as the gold standard. It is suggested the Kappa value be interpreted as follows: values ≤0 as indicating no agreement and 0.01–0.20 as none to slight, 0.21–0.40 as fair, 0.41–0.60 as moderate, 0.61–0.80 as substantial, and 0.81–1.00 as almost perfect agreement.

## Results

### qPCR optimisation

PhaHV-1 qPCRs consistently resulted in melt temperatures of 81–81.5°C (S2a Fig in S1 File) and efficiencies ranging from 90.1–106.7%. The limit of detection was determined as 12 copies per reaction (corresponding to a Ct-value of 37.5, S2b Fig and S1 Table in S1 File), with a quantifiable range over six 10-fold serial dilutions. Target specificity of the newly designed primers was confirmed by successful amplification of PhaHV-1 DNA and no amplification for other bacterial or viral DNA tested. BLAST results from sequenced PCR products all exclusively aligned to phascolarctid gammaherpesvirus 1 *dpol* with 98–100% sequence identity.

### Determining best sample for evaluating PhaHV-1 load/detection via qPCR

Of the 47 koalas tested using paired DNA extracts from swab samples, 27 (57%) were positive for PhaHV-1 as indicated by at least one anatomical site and 20 koalas were negative at all anatomical sites (S4 Table in S1 File). Initial investigation of optimal sample type for detection of PhaHV-1 infection indicated that of 47 paired sets of DNA extracted from oropharyngeal (Oro/Phx) and urogenital (UGT) swab samples, 12 koalas were positive for PhaHV-1 at both and 20 were negative at both. Ten koalas were positive at Oro/Phx and negative at UGT swabs and five koalas were negative at Oro/Phx and positive at UGT swabs. Of the six ocular swab results available from these animals, five were in agreement to their matched UGT and Oro/Phx sample results (1 positive and 4 negative) and one ocular swab was negative while the paired UGT and Oro/Phx samples were positive.

Of the 29 scat samples, seven were positive for PhaHV-1 in agreement with at least one swab sample from the same animal. There were no false positives results from scat samples, i.e., all scats were negative from 13 koalas whose swabs were also negative. There were nine scat samples that were negative where at least one swab sample from the same koala was positive. This corresponds to a specificity of 100% and a sensitivity of 44% when assessing this sample type.

### PhaHV-1 LAMP assay design, sensitivity and specificity

During preliminary assay design, we selected three primers sets, one of each designed by the three different LAMP primer tools. Although all three primer sets amplified PhaHV-1 DNA and no non-specific amplification was observed, primers designed with a GLAPD tool were selected for further assay optimisation and testing. This primer set was most efficient in achieving amplification of low DNA copy number template (added as 5 or 1 μL in reaction) in comparison to the other two primer sets (ie. the remaining sets had low amplification efficiency and were only able to amplify high DNA copy samples).

The limit of detection of the PhaHV-1 LAMP assay was conservatively estimated to be 10 genome copies/reaction (5/5 replicates, 100% detection) (S2 Table in S1 File). Similarly to the qPCR assay, target specificity of the newly designed primer set was confirmed by successful amplification of PhaHV-1 DNA, tested individually and/or as a mixture with other non-targeted DNA, and no amplification for other bacterial or viral DNA tested (S3 Table in S1 File).

### PhaHV-1 detection in DNA and rapidly processed clinical swabs using isothermal assays

Comparisons between the qPCR assay and the LAMP using 70 DNA samples, demonstrated 94.29% overall agreement between the two assays, with Kappa values of 0.8113 indicating an almost perfect agreement (Table 3, S5 Table in S1 File). Discrepant results were noted for four samples, where sample K4 OCC was positive by LAMP but negative by qPCR, and remaining three samples (K5 REC, K8 UGT, and K30 OCC) were negative by LAMP but positive by qPCR assay (S5 Table in S1 File).

**Table 3:** Comparison of PhaHV-1 LAMP assay to the PhaHV-1 reference qPCR assay when testing DNA samples.

Test	Reference PhaHV-1 qPCR		
**PhaHV-1 LAMP**	Positive	Negative	Total
Positive	11	1	12
Negative	3	55	58
Total	14	56	70
Overall agreement^a^	94.29% (95% CI: 0.8601–0.9842)		
	84.62% PA (95% CI: 0.6972–0.9951)		
	96.49% NA (95% CI: 0.9305–0.9993)		
McNemar’s Chi sq (pChi sq)	0.25 (0.6171)		
Kappa (0.95 CI, pKappa)	0.8113 (0.6336–0.9891; 0)		

^a^ positive agreement (PA) and negative agreement (NA) are also outlined.

For the 36 rapidly processed swab suspensions, the overall agreement between the PhaHV-1 LAMP and the qPCR assay was 94.44% with Kappa values of 0.7118 indicating substantial agreement (Table 4). Discrepant results were noted for two samples, where sample K6 REC was highly positive by LAMP but negative by qPCR, and sample K16 E was negative by LAMP but weakly positive by qPCR with an average Ct of 34.56 (S6 Table in S1 File).

**Table 4:** Comparison of the PhaHV-1 LAMP testing of rapidly processed swabs to the PhaHV-1 reference qPCR testing of DNA extracted from the same swab suspensions.

Test	Reference PhaHV-1 qPCR		
**PhaHV-1 LAMP**	Positive	Negative	Total
Positive	3	1	4
Negative	1	31	32
Total	4	32	36
Overall agreement^a^	94.44 (95% CI: 0.8134–0.9932)		
	75% PA (95% CI: 0.4145–1.0855)		
	96.88% NA (95% CI: 0.9255–1.0120)		
McNemar’s Chi sq (pChi sq)	0.5 (0.4795)		
Kappa (0.95 CI, pKappa)	0.7188 (95% CI: 0.3497–1.0878; 0)		

### Testing for inhibition of amplification in rapidly processed samples

No PhaHV-1 was detected by LAMP in three of 14 “spiked” rapidly processed swab suspensions (samples K1 REC, K2 REC, and K16 UGT; S6 Table in S1 File), indicating the potential presence of inhibitors in these samples. When testing the DNA extracted from the same “spiked” swabs using qPCR, only one sample (K2 REC) was inhibited, with no PhaHV-1 DNA detected; PhaHV-1 DNA was detected in the DNA extracted from the remaining two “spiked” swab samples inhibited in LAMP (K1 REC and K16 UGT), but with late amplification (S6 Table in S1 File).

## Discussion

We have developed and optimised two new assays for detection of PhaHV-1 in koalas. The qPCR assay provides a high-throughput, specific, and sensitive diagnostic test that can quantify viral load; to be used in research settings alongside existing assays for chlamydia and KoRV diagnostics. This will allow further research on the distribution and prevalence of PhaHV-1 infection as well as increasing our understanding of the dynamics of co-infections. The LAMP assay provides a robust, rapid, point-of-care test, with applications in clinical and field settings. This will aid rapid diagnostics to guide quarantine of koalas in care. The use of both assays will enable vital research into the distribution, prevalence and significance of PhaHV-1 infection in the endangered koala populations of NSW, Qld and the ACT, where there is currently no information on this pathogen.

We recommend multiple samples from different anatomical sites from the same animal be used to maximise sensitivity of PhaHV-1 detection. In our study, PhaHV-1 detection by qPCR was slightly more sensitive in oropharyngeal swabs compared to urogenital swabs. Studies are equivocal as to whether detection is more likely in oropharyngeal or urogenital swabs [10, 11], and larger sample sizes may be needed to resolve this question. DNA extracted from scat was not a reliable sample for PhaHV-1 detection using our assay. We cannot conclusively determine whether the assays we have developed are detecting all infected animals or only lytic infections, as the virus in latent form may be present in circulating B cells [14]. Regardless, the ability to rapidly quantify viral load will advance research into the pathogenesis of PhaHV-1 infection, its relationship to stress and disease and its significance amongst other co-infections.

In this study, we also demonstrated the successful use of rapid isothermal assay of rapidly processed swabs to detect PhaHV-1 infections, supporting the proof of concept that rapid isothermal diagnostics can be applied at the koala and/or other veterinary clinical setting [33, 35, 36]. Similar to *Chlamydia* isothermal assays [26, 27], the PhaHV-1 isothermal assay performed in the Genie III fluorometer provides straightforward interpretation of results, with positive results being distinctly characterised by amplification time and specific melt curves. Although LAMP is considered robust to PCR inhibition, the presence of PCR inhibitors, from the sample itself or introduced during sample processing, can produce false-negative results [37] and we found this to be the case. We observed better congruence between LAMP and the reference qPCR when testing kit-extracted DNA samples; reduced sensitivity and increased inhibition of LAMP was observed when testing rapidly processed swab samples. For example, two rectal (K1 REC and K2 REC) and a UGT (K16 UGT) swabs perhaps contained inhibitors and did not amplify in LAMP and qPCR (K2 REC) or produced late amplification (K1 RE, K16 UGT) on qPCR after DNA extraction. Nevertheless, rapid swab processing and crude DNA extraction can be improved using commercially available DNA lysis buffers and, as recommended above, swabs from multiple anatomical sites from the same animal should be tested. In this study, we only tested previously collected mucosal swabs, a further investigation testing fresh swabs, comparing sample storage methods and improved sample lysis is required to fully harness the potential of this technology.

The assays we have developed will support the National Koala Monitoring Program and state-based monitoring programs, translocation risk assessment, welfare and biosecurity in rehabilitation, as well as implementation of the National Koala Disease Risk Assessment [3]. Future work can develop rapid diagnostics for PhaHV-2, but this will first require more sequence information. The PhaHV-1 assays we have developed are now being made available in research and clinical settings, enabling collaborative research across institutions to better define the prevalence and impacts of PhaHV-1 infections.

## Supporting information

S1 File Contains all supporting tables and figures. (DOCX)

## References

[1] Australian Government Department of Climate Change EtEaW. https://www.environment.gov.au/cgi-bin/sprat/public/publicthreatenedlist.pl?wanted=fauna

[2] Grogan LF, Peel AJ, Kerlin D, Ellis W, Jones D, Hero J-M, et al. Is disease a major causal factor in declines? An evidence framework and case study on koala chlamydiosis. Biological Conservation. 2018;221:334–44.

[3] Vitali SD RA, Jakob-Hoff RM, Stephenson TL, Holz PH and Higgins DP. National Koala Disease Risk Analysis Report. University of Sydney; 2022.

[4] Quigley BL, Timms P. Helping koalas battle disease–Recent advances in Chlamydia and koala retrovirus (KoRV) disease understanding and treatment in koalas. FEMS Microbiology Reviews. 2020;44(5):583–605. doi: 10.1093/femsre/fuaa024 32556174 PMC8600735

[5] Robbins A, Loader J, Timms P, Hanger J. Optimising the short and long-term clinical outcomes for koalas (*Phascolarctos cinereus*) during treatment for chlamydial infection and disease. PLoS One. 2018;13(12):e0209679. 30589897 10.1371/journal.pone.0209679 PMC6307739

[6] Tarlinton R, Meers J, Hanger J, Young P. Real-time reverse transcriptase PCR for the endogenous koala retrovirus reveals an association between plasma viral load and neoplastic disease in koalas. Journal of General Virology. 2005;86(3):783–7. doi: 10.1099/vir.0.80547-0 15722540

[7] Xu W, Stadler CK, Gorman K, Jensen N, Kim D, Zheng H, et al. An exogenous retrovirus isolated from koalas with malignant neoplasias in a US zoo. Proceedings of the National Academy of Sciences. 2013;110(28):11547–52. 10.1073/pnas.1304704110 PMC3710800 23798387

[8] Vaz P, Whiteley P, Wilks C, Duignan P, Ficorilli N, Gilkerson J, et al. Detection of a novel gammaherpesvirus in koalas (*Phascolarctos cinereus*). Journal of Wildlife Diseases. 2011;47(3):787–91. 21719855 10.7589/0090-3558-47.3.787

[9] Vaz P, Whiteley PL, Wilks CR, Browning GF, Gilkerson JR, Ficorilli N, et al. Detection of a second novel gammaherpesvirus in a free-ranging koala (*Phascolarctos cinereus*). Journal of Wildlife Diseases. 2012;48(1):226–9. 22247398 10.7589/0090-3558-48.1.226

[10] Vaz P, Legione A, Hartley C, Devlin J. Detection and differentiation of two koala gammaherpesviruses by use of high-resolution melt (HRM) analysis reveals differences in viral prevalence and clinical associations in a large study of free-ranging koalas. Journal of Clinical Microbiology. 2019;57(3):e01478–18. doi: 10.1128/JCM.01478-18 30626662 PMC6425191

[11] Kasimov V, Stephenson T, Speight N, Chaber A-L, Boardman W, Easther R, et al. Identification and prevalence of phascolarctid gammaherpesvirus Types 1 and 2 in South Australian koala populations. Viruses. 2020;12(9):948. doi: 10.3390/v12090948 32867109 PMC7552032

[12] Matthews R. Classification and nomenclature of viruses. Intervirology. 1982;17(1):199. 10.1159/000149278 6811498

[13] Drummer H, Reubel G, Studdert M. Equine gammaherpesvirus 2 (EHV2) is latent in B lymphocytes. Archives of virology. 1996;141(3):495–504. doi: 10.1007/BF01718313 8645091

[14] Flaño E, Kim I-J, Woodland DL, Blackman MA. γ-Herpesvirus latency is preferentially maintained in splenic germinal center and memory B cells. The Journal of Experimental Medicine. 2002;196(10):1363–72. 12438427 10.1084/jem.20020890 PMC2193987

[15] Fujiwara S, Nakamura H. Animal models for gammaherpesvirus infections: recent development in the analysis of virus-induced pathogenesis. Pathogens. 2020;9(2):116. doi: 10.3390/pathogens9020116 32059472 PMC7167833

[16] Ackermann M. Pathogenesis of gammaherpesvirus infections. Veterinary Microbiology. 2006;113(3–4):211–22. doi: 10.1016/j.vetmic.2005.11.008 16332416

[17] Vaz PK, Hartley CA, Lee S-Y, Sansom FM, Adams TE, Stalder K, et al. Koala and wombat gammaherpesviruses encode the first known viral NTPDase homologs and are phylogenetically divergent from all known gammaherpesviruses. Journal of Virology. 2018;93(6):e01404–18. 10.1128/JVI.01404-18 PMC6401436 30567986

[18] Matar CG, Anthony NR, O’Flaherty BM, Jacobs NT, Priyamvada L, Engwerda CR, et al. Gammaherpesvirus co-infection with malaria suppresses anti-parasitic humoral immunity. PLoS Pathogens. 2015;11(5):e1004858. doi: 10.1371/journal.ppat.1004858 25996913 PMC4440701

[19] Prusty BK, Böhme L, Bergmann B, Siegl C, Krause E, Mehlitz A, et al. Imbalanced oxidative stress causes chlamydial persistence during non-productive human herpes virus co-infection. PLoS One. 2012;7:e47427. doi: 10.1371/journal.pone.0047427 23077614 PMC3471814

[20] Prusty BK, Siegl C, Hauck P, Hain J, Korhonen SJ, Hiltunen-Back E, et al. Chlamydia trachomatis infection induces replication of latent HHV-6. PLoS One. 2013;8(4):e61400. doi: 10.1371/journal.pone.0061400 23620749 PMC3631192

[21] Vanover J, Sun J, Deka S, Kintner J, Duffourc MM, Schoborg RV. Herpes simplex virus co-infection-induced Chlamydia trachomatis persistence is not mediated by any known persistence inducer or anti-chlamydial pathway. Microbiology. 2008;154(3):971–8. doi: 10.1099/mic.0.2007/012161-0 18310043

[22] Caselli E, Borghi A, Maritati M, Gafà R, Lanza G, Di Luca D, et al. Relapses of primary cutaneous anaplastic large-cell lymphoma in a female immunocompetent patient with persistent chlamydophila pneumoniae and human herpesvirus 8 infection. Infectious Agents and Cancer. 2016;11:1–6. 27382410 10.1186/s13027-016-0079-4 PMC4932675

[23] Stalder K, Vaz PK, Gilkerson JR, Baker R, Whiteley P, Ficorilli N, et al. Prevalence and clinical significance of herpesvirus infection in populations of Australian marsupials. PLoS One. 2015;10(7):e0133807. doi: 10.1371/journal.pone.0133807 26222660 PMC4519311

[24] Mackay IM, Arden KE, Nitsche A. Real-time PCR in virology. Nucleic Acids Research. 2002;30(6):1292–305. doi: 10.1093/nar/30.6.1292 11884626 PMC101343

[25] Notomi T, Okayama H, Masubuchi H, Yonekawa T, Watanabe K, Amino N, et al. Loop-mediated isothermal amplification of DNA. Nucleic Acids Research. 2000;28(12):e63–e. doi: 10.1093/nar/28.12.e63 10871386 PMC102748

[26] Jelocnik M, Islam MM, Madden D, Jenkins C, Branley J, Carver S, et al. Development and evaluation of rapid novel isothermal amplification assays for important veterinary pathogens: *Chlamydia psittac*i and *Chlamydia pecorum*. PeerJ. 2017;5:e3799. 28929022 10.7717/peerj.3799 PMC5592900

[27] Hulse LS, McDonald S, Johnston SD, Beagley KW. Rapid point-of-care diagnostics for the detection of Chlamydia pecorum in koalas (*Phascolarctos cinereus*) using loop-mediated isothermal amplification without nucleic acid purification. MicrobiologyOpen. 2019;8(12):e916. 31419041 10.1002/mbo3.916 PMC6925175

[28] Untergasser A, Nijveen H, Rao X, Bisseling T, Geurts R, Leunissen JA. Primer3Plus, an enhanced web interface to Primer3. Nucleic Acids Research. 2007;35(suppl_2):W71–W4. doi: 10.1093/nar/gkm306 17485472 PMC1933133

[29] Owczarzy R, Tataurov AV, Wu Y, Manthey JA, McQuisten KA, Almabrazi HG, et al. IDT SciTools: a suite for analysis and design of nucleic acid oligomers. Nucleic Acids Research. 2008;36(suppl_2):W163–W9. doi: 10.1093/nar/gkn198 18440976 PMC2447751

[30] Altschul SF, Gish W, Miller W, Myers EW, Lipman DJ. Basic local alignment search tool. Journal of Molecular Biology. 1990;215(3):403–10. doi: 10.1016/S0022-2836(05)80360-2 2231712

[31] Hulse LS, Hickey D, Mitchell JM, Beagley KW, Ellis W, Johnston SD. Development and application of two multiplex real-time PCR assays for detection and speciation of bacterial pathogens in the koala. Journal of Veterinary Diagnostic Investigation. 2018;30(4):523–9. doi: 10.1177/1040638718770490 29629645 PMC6505923

[32] Jia B, Li X, Liu W, Lu C, Lu X, Ma L, et al. GLAPD: whole genome based LAMP primer design for a set of target genomes. Frontiers in Microbiology. 2019;10:2860. doi: 10.3389/fmicb.2019.02860 31921040 PMC6923652

[33] Clune T, Anstey S, Kasimov V, Jacobson C, Jelocnik M. Real-time fluorometric isothermal LAMP assay for detection of *Chlamydia pecorum* in rapidly processed ovine abortion samples: a Veterinary practitioner’s perspective. Pathogens. 2021;10(9):1157. 34578188 10.3390/pathogens10091157 PMC8470028

[34] Sergeant E. Epitools epidemiological calculators 2018 [Available from: http://epitools.ausvet.com.au.

[35] Jelocnik M, Nyari S, Anstey S, Playford N, Fraser TA, Mitchell K, et al. Real-time fluorometric and end-point colorimetric isothermal assays for detection of equine pathogens *C*. *psittaci* and equine herpes virus 1: validation, comparison and application at the point of care. BMC veterinary Research. 2021;17(1):1–15. 34412635 10.1186/s12917-021-02986-8 PMC8375077

[36] Mansour SM, Ali H, Chase CC, Cepica A. Loop-mediated isothermal amplification for diagnosis of 18 World Organization for Animal Health (OIE) notifiable viral diseases of ruminants, swine and poultry. Animal Health Research Reviews. 2015;16(2):89–106. doi: 10.1017/S1466252315000018 25900363

[37] Schrader C, Schielke A, Ellerbroek L, Johne R. PCR inhibitors–occurrence, properties and removal. Journal of Applied Microbiology. 2012;113(5):1014–26. doi: 10.1111/j.1365-2672.2012.05384.x 22747964

