# Supplementary material for: Development of diagnostic and point of care assays for a gammaherpesvirus infecting koalas
Source: PLoS One. 2023 Jun 1;18(6):e0286407. doi: 10.1371/journal.pone.0286407 (PMC10234535; doi:10.1371/journal.pone.0286407)
Supplement: S1 File — (DOCX) [file pone.0286407.s001.docx]

Supplementary material to: Development of diagnostic and point of care assays for a gammaherpesvirus infecting koalas.

Belinda R. Wright^1¶*^, Martina Jelocnik^2¶^, Andrea Casteriano^1^, Yasmine S.S. Muir^1^, Alistair R. Legione^3^, Paola K. Vaz^3^, Joanne M. Devlin^3^, Damien P. Higgins^1^

1. Koala Health Hub, Sydney School of Veterinary Science, University of Sydney, Camperdown, 2006, New South Wales, Australia

2. Centre for Bioinnovation, University of The Sunshine Coast, Sippy Downs, 4557, Queensland, Australia

3. Asia Pacific Centre for Animal Health, Melbourne Veterinary School, Faculty of Science, University of Melbourne, Parkville, 3010, Victoria, Australia

¶ These authors contributed equally

* Corresponding author

Email: belinda.wright@sydney.edu.au

S1 Table. qPCR sensitivity determined by serial dilutions of PhaHV-1 synthetic positive control. See S2a Fig. for resulting melt curves.

| *Copies/reaction* | *No. replicates* | *Average Ct-value* | | *No. Positives* | *% Positive* | | *Probit Value* |
| --- | --- | --- | --- | --- | --- | --- | --- |
| 100,000,000 | 4 | | 13.43 | 4 | 100 | 8.09 | |
| 10,000,000 | 4 | | 17.04 | 4 | 100 | 8.09 | |
| 1,000,000 | 8 | | 20.27 | 8 | 100 | 8.09 | |
| 100,000 | 8 | | 23.51 | 8 | 100 | 8.09 | |
| 10,000 | 4 | | 27.13 | 4 | 100 | 8.09 | |
| 1000 | 10 | | 30.49 | 10 | 100 | 8.09 | |
| 100 | 10 | | 34.06 | 10 | 100 | 8.09 | |
| 50 | 10 | | 35.21 | 10 | 100 | 8.09 | |
| 25 | 10 | | 36.18 | 10 | 100 | 8.09 | |
| 10 | 10 | | 37.66 | 7 | 70 | 5.52 | |
| 5 | 10 | | 38.1 | 7 | 70 | 5.52 | |
| 1 | 10 | | 39.2 | 1 | 10 | 3.72 | |

S2 Table. PhaHV-1 isothermal assay sensitivity from serial dilutions of synthetic positive control.

| *Replicate* | *DNA copy number in rxn* | *PhaHV-1 set* | | *Mean time (min:sec); melt (°C) (SD) and % detection* |
| --- | --- | --- | --- | --- |
|  |  | *Time (min:sec)* | *Melt (°C)* |  |
| 1 | 500 copies | 14:45 | 87.82 | 13:40 (±1:07); 87.89 (0.08); 3/3 (100%) |
| 2 |  | 13:45 | 87.97 |  |
| 3 |  | 12:30 | 87.87 |  |
| 1 | 100 copies | 17:30 | 87.77 | 16:24 (±1:33); 87.92 (0.20); 5/5 (100%) |
| 2 |  | 17:00 | 87.77 |  |
| 3 |  | 14:45 | 88.25 |  |
| 4 |  | 18:00 | 87.86 |  |
| 5 |  | 14:45 | 87.97 |  |
| 1 | 50 copies | 20:00 | 87.77 | 18:12 (±1:32); 87.81 (0.11); 5/5 (100%) |
| 2 |  | 19:30 | 87.72 |  |
| 3 |  | 17:45 | 87.97 |  |
| 4 |  | 16:15 | 87.72 |  |
| 5 |  | 17:30 | 87.87 |  |
| 1 | 10 copies | 18:00 | 87.72 | 20:39 (±2:75); 87.75 (0.03); 5/5 (100%) |
| 2 |  | 22:00 | 87.77 |  |
| 3 |  | 22:30 | 87.72 |  |
| 4 |  | 19:00 | 87.76 |  |
| 5 |  | 21:45 | 87.78 |  |
| 1 | 1 copy | 26:00 | 87.92 | 27:05 (+- 1:03); 87.82 (0.05); 3/5 (60%) |
| 2 |  | 27:10 | 87.83 |  |
| 3 |  | 28:00 | 87.87 |  |
| 4 |  | NA | NA |  |
| 5 |  | NA | NA |  |
| 1 | 0 copies | NA NA | | NA |
| 2 |  |  |  |  |
| 3 |  |  |  |  |
| 4 |  |  |  |  |
| 5 |  |  |  |  |

NA: no amplification.

S3 Table. LAMP target specificity.

| *Samples* | *LAMP primer set from this study, denoting time to amplify (min:ss) and melt (°C)* |
| --- | --- |
| PhaHV1 DNA 1^*^ | 12:00; 87.85 |
| PhaHV1 DNA 2^*^ | 14:15; 87.87 |
| PhaHV2 DNA 1 | NA |
| PhaHV2 DNA 2 | NA |
| Macropodid HV (MaHV) DNA^ʈ^ | NA |
| *C. pecorum* Marsbar_2018 DNA | NA |
| Mixed *C. pecorum*/KORV DNA | NA |
| Mixed PhaHV1/ PhaHV2 DNA | 13:45; 87.76 |
| Mixed PhaHV1/ MaHV DNA | 13:50; 87.81 |

NA: no amplification. *PhaHV1 strain used is 36M/11, ʈ MaHV1 strain used is V3076/08. For all testing, 5µl template was added in the reaction as recommended by the manufacturer.

S4 Table. qPCR results for paired DNA samples from differing anatomical sites. O/Phx, oropharangeal swab; UGT, urogenital swab.

| *Sample ID* | *Collection date* | *Sample type* | *β actin Ct mean* | *β actin Ct S.D.* | *PhaHV-1 Ct mean* | *PhaHV-1 Ct S.D.* | *PhaHV-1 result* |
| --- | --- | --- | --- | --- | --- | --- | --- |
| 109085 | 20/09/2021 | Ocular | 24.52 | 0.02 |  |  | neg |
| 109085 | 20/09/2021 | O/Phx | 22.36 | 0.15 | 31.93 | 0.01 | pos |
| 109085 | 20/09/2021 | UGT | 19.17 | 0.01 | 35.50 | 0.11 | pos |
| 109085 | 20/09/2021 | scat | 18.61 | 0.13 | 27.65 | 0.01 | pos |
| 109090 | 20/09/2021 | Ocular | 16.73 | 0.09 | 34.79 | 0.30 | pos |
| 109090 | 20/09/2021 | O/Phx | 22.92 | 0.15 | 33.45 | 0.03 | pos |
| 109090 | 20/09/2021 | UGT | 22.36 | 0.06 | 32.82 | 0.09 | pos |
| 109090 | 20/09/2021 | scat | 20.57 | 0.00 | 24.01 | 0.07 | pos |
| 109112 | 21/09/2021 | Ocular | 23.32 | 0.08 |  |  | neg |
| 109112 | 21/09/2021 | O/Phx | 24.12 | 0.08 |  |  | neg |
| 109112 | 21/09/2021 | UGT | 26.46 | 0.07 |  |  | neg |
| 109113 | 21/09/2021 | Ocular | 24.54 | 0.16 |  |  | neg |
| 109113 | 21/09/2021 | O/Phx | 26.30 | 0.06 |  |  | neg |
| 109113 | 21/09/2021 | UGT | 28.12 | 0.00 |  |  | neg |
| 109137 | 22/09/2021 | Ocular | 26.97 | 0.16 |  |  | neg |
| 109137 | 22/09/2021 | UGT | 23.25 | 0.02 |  |  | neg |
| 109137 | 22/09/2021 | O/Phx | 28.25 | 0.11 |  |  | neg |
| 109137 | 22/09/2021 | scat | 21.31 | 0.06 |  |  | neg |
| 109141 | 22/09/2021 | Ocular | 27.02 | 0.02 |  |  | neg |
| 109141 | 24/09/2021 | O/Phx | 25.64 | 0.02 | 39.40 |  | neg |
| 109141 | 23/09/2021 | UGT | 23.72 | 0.13 |  |  | neg |
| 109141 | 22/09/2021 | scat | 29.00 | 0.11 | 39.23 |  | neg |
| 109253 | 24/09/2021 | O/Phx | 25.68 | 0.11 |  |  | neg |
| 109253 | 24/09/2021 | UGT | 25.58 | 0.04 |  |  | neg |
| 109253 | 24/09/2021 | scat | 24.94 | 0.17 | 39.25 | 0.25 | neg |
| 109425 | 29/09/2021 | O/Phx | 34.99 | 1.94 |  |  | neg |
| 109425 | 29/09/2021 | UGT | 23.50 | 0.00 |  |  | neg |
| 109425 | 29/09/2021 | scat | 25.16 | 0.09 |  |  | neg |
| 109708 | 7/10/2021 | O/Phx | 22.66 | 0.06 |  |  | neg |
| 109708 | 7/10/2021 | UGT | 23.50 | 0.02 |  |  | neg |
| 109708 | 7/10/2021 | scat | 24.47 | 0.05 | 39.61 |  | neg |
| 109724 | 7/10/2021 | O/Phx | 21.27 | 0.12 | 25.60 | 0.19 | pos |
| 109724 | 7/10/2021 | UGT | 17.65 | 0.12 | 26.72 | 0.03 | pos |
| 109724 | 7/10/2021 | scat | 22.09 | 0.09 | 29.86 | 0.01 | pos |
| 109738 | 8/10/2021 | O/Phx | 30.67 | 0.24 |  |  | neg |
| 109738 | 8/10/2021 | UGT | 17.40 | 0.05 | 34.83 | 0.05 | pos |
| 109738 | 8/10/2021 | scat | 27.75 | 0.12 |  |  | neg |
| 110282 | 21/10/2021 | O/Phx | 24.91 | 0.16 |  |  | neg |
| 110282 | 21/10/2021 | UGT | 24.05 | 0.06 |  |  | neg |
| 110282 | 21/10/2021 | scat | 21.66 | 0.06 |  |  | neg |
| 110284 | 21/10/2022 | O/Phx | 17.67 | 0.01 | 27.11 | 0.16 | pos |
| 110284 | 21/10/2022 | UGT | 20.18 | 0.05 | 31.05 | 0.04 | pos |
| 110284 | 21/10/2021 | scat | 21.18 | 0.07 | 29.01 | 0.22 | pos |
| 110301 | 22/10/2021 | O/Phx | 24.72 | 0.03 | 36.96 | 0.03 | pos |
| 110301 | 22/10/2021 | UGT | 18.46 | 0.02 | 35.44 | 0.12 | pos |
| 110301 | 22/10/2021 | scat | 20.99 | 0.03 | 34.65 | 0.50 | pos |
| 110396 | 24/10/2022 | O/Phx | 21.02 | 0.19 | 30.26 | 0.02 | pos |
| 110396 | 24/10/2022 | UGT | 23.45 | 0.14 |  |  | neg |
| 110396 | 24/10/2021 | scat | 25.18 | 0.15 |  |  | neg |
| 110476 | 25/10/2022 | O/Phx | 21.23 | 0.06 | 29.18 | 0.03 | pos |
| 110476 | 25/10/2022 | UGT | 23.26 | 0.26 |  |  | neg |
| 110476 | 25/10/2021 | scat | 22.47 | 0.00 | 38.66 | 0.51 | neg |
| 110505 | 26/10/2022 | O/Phx | 22.05 | 0.00 |  |  | neg |
| 110505 | 26/10/2022 | UGT | 17.74 | 0.20 |  |  | neg |
| 110505 | 26/10/2021 | scat | 20.21 | 0.01 | 38.65 | 0.53 | neg |
| 110745 | 6/11/2021 | O/Phx | 25.38 | 0.17 |  |  | neg |
| 110745 | 6/11/2021 | UGT | 21.58 | 0.02 |  |  | neg |
| 110745 | 1/11/2021 | scat | 23.71 | 0.19 | 38.67 | 1.31 | neg |
| 110882 | 8/11/2021 | O/Phx | 28.51 | 0.48 |  |  | neg |
| 110882 | 8/11/2021 | UGT | 17.67 | 0.35 |  |  | neg |
| 110882 | 8/11/2021 | scat | 20.60 | 0.03 | 39.13 |  | neg |
| 111067 | 9/11/2021 | O/Phx | 31.14 | 0.25 |  |  | neg |
| 111067 | 9/11/2021 | UGT | 20.87 | 0.06 |  |  | neg |
| 111067 | 9/11/2021 | scat | 22.17 | 0.10 |  |  | neg |
| 111075 | 9/11/2021 | O/Phx | 25.71 | 0.03 |  |  | neg |
| 111075 | 9/11/2021 | UGT | 20.88 | 0.08 |  |  | neg |
| 111075 | 9/11/2021 | scat | 24.87 | 0.50 |  |  | neg |
| 111097 | 10/11/2021 | O/Phx | 22.83 | 2.20 | 29.89 | 0.26 | pos |
| 111097 | 10/11/2021 | UGT | 18.51 | 0.02 | 24.86 | 0.07 | pos |
| 111097 | 10/11/2021 | scat | 20.77 | 0.02 |  |  | neg |
| 111212 | 11/11/2021 | O/Phx | 22.23 | 0.05 | 32.88 | 0.01 | pos |
| 111212 | 11/11/2021 | UGT | 22.67 | 0.00 |  |  | neg |
| 111212 | 11/11/2021 | scat | 22.73 | 0.12 |  |  | neg |
| 112243 | 9/12/2021 | O/Phx | 23.01 | 0.12 | 31.09 | 0.04 | pos |
| 112243 | 9/12/2021 | UGT | 21.03 | 0.25 | 25.01 | 0.00 | pos |
| 112243 | 9/12/2021 | scat | 22.82 | 0.06 | 34.19 | 0.03 | pos |
| 2021-141 | 8/03/2022 | O/Phx | 24.04 | 0.14 | 34.97 | 0.13 | pos |
| 2021-141 | 8/03/2022 | UGT | 19.44 | 0.12 |  |  | neg |
| 2021-141 | 8/03/2022 | scat | 19.48 | 0.12 | 38.00 | 0.09 | neg |
| 2020-197 | 7/03/2022 | O/Phx | 26.24 | 0.13 |  |  | neg |
| 2020-197 | 7/03/2022 | UGT | 18.16 | 0.00 |  |  | neg |
| 2020-197 | 8/03/2022 | scat | 23.82 |  |  |  | neg |
| 2022-015 | 27/01/2022 | O/Phx | 21.22 | 0.06 | 29.23 | 0.04 | pos |
| 2022-015 | 27/01/2022 | UGT | 18.64 | 0.04 | 31.33 | 0.09 | pos |
| 2022-015 | 28/02/2022 | scat | 22.43 | 0.07 | 38.18 | 0.26 | neg |
| 2022-016 | 28/01/2022 | O/Phx | 22.09 | 0.08 | 32.45 | 0.12 | pos |
| 2022-016 | 28/01/2022 | UGT | 22.35 | 0.09 |  |  | neg |
| 2022-016 | 28/01/2022 | scat | 20.08 | 0.08 | 39.69 |  | neg |
| 2022-025 | 15/02/2022 | O/Phx | 20.39 | 0.03 | 28.53 | 0.01 | pos |
| 2022-025 | 15/02/2022 | UGT | 20.98 | 0.12 |  |  | neg |
| 2022-025 | 15/02/2022 | scat | 22.19 | 0.00 | 39.37 | 0.29 | neg |
| 2022-007 | 12/01/2022 | O/Phx | 25.06 | 0.30 |  |  | neg |
| 2022-007 | 12/01/2022 | UGT | 21.52 | 0.15 |  |  | neg |
| 2022-007 | 12/01/2022 | scat | 26.11 | 0.09 | 37.83 |  | neg |
| 2202057 | 7/02/2022 | O/Phx | 25.38 | 0.06 | 33.68 | 0.13 | pos |
| 2202057 | 7/02/2022 | UGT | 18.97 | 0.12 | 35.21 | 0.10 | pos |
| 2202057 | 7/02/2022 | scat | 24.14 |  | 33.33 | 0.05 | pos |
| 109247 | 24/09/2021 | O/Phx | 21.74 | 0.02 | 27.15 | 0.03 | pos |
| 109247 | 24/09/2021 | UGT | 21.70 | 0.04 |  |  | neg |
| 109461 | 30/09/2021 | O/Phx | 23.12 | 0.08 | 36.35 | 0.22 | pos |
| 109461 | 30/09/2021 | UGT | 38.09 | 0.38 |  |  | neg |
| 109480 | 1/10/2021 | O/Phx | 27.64 | 0.09 |  |  | neg |
| 109480 | 1/10/2021 | UGT | 23.25 | 0.24 |  |  | neg |
| 110044 | 16/10/2021 | O/Phx | 22.15 | 0.11 |  |  | neg |
| 110044 | 16/10/2021 | UGT | 24.98 | 0.12 |  |  | neg |
| 110220 | 19/10/2021 | O/Phx | 26.35 | 0.04 |  |  | neg |
| 110220 | 19/10/2021 | UGT | 24.70 | 0.20 | 35.92 | 0.23 | pos |
| 110327 | 22/10/2022 | O/Phx | 24.80 | 0.23 | 31.90 | 0.20 | pos |
| 110327 | 22/10/2022 | UGT | 22.12 | 0.06 |  |  | neg |
| 112058 | 17/12/2021 | O/Phx | 23.56 | 0.03 | 32.88 | 0.14 | pos |
| 112058 | 17/12/2022 | UGT | 19.26 | 0.09 |  |  | neg |
| 112071 | 17/12/2021 | O/Phx | 19.87 | 0.07 | 27.68 | 0.09 | pos |
| 112071 | 17/12/2022 | UGT | 17.10 | 0.12 | 34.71 | 0.09 | pos |
| 112386 | 17/12/2021 | O/Phx | 27.60 | 0.03 |  |  | neg |
| 112386 | 17/12/2022 | UGT | 20.30 | 0.05 |  |  | neg |
| 2021-137 | 9/04/2022 | O/Phx | 24.34 | 0.13 | 30.62 | 0.18 | pos |
| 2021-137 | 9/04/2022 | UGT | 19.23 | 0.02 | 28.44 | 0.03 | pos |
| 2022-002 | 18/02/2022 | O/Phx | 29.49 | 0.11 |  |  | neg |
| 2022-002 | 18/02/2022 | UGT | 20.65 | 0.12 | 28.99 | 0.02 | pos |
| 2022-008 | 13/01/2022 | O/Phx | 20.72 | 0.05 | 33.16 | 0.15 | pos |
| 2022-008 | 13/01/2022 | UGT | 17.45 | 0.06 | 34.81 | 0.15 | pos |
| 2022-014 | 25/01/2022 | O/Phx | 33.94 | 1.60 |  |  | neg |
| 2022-014 | 25/01/2022 | UGT | 17.40 | 0.05 | 30.65 | 0.11 | pos |
| 2022-018 | 8/02/2022 | O/Phx | 33.48 | 1.22 |  |  | neg |
| 2022-018 | 8/02/2022 | UGT | 16.60 | 0.07 |  |  | neg |
| 2022-028 | 25/02/2022 | O/Phx | 30.85 | 0.27 |  |  | neg |
| 2022-028 | 25/02/2022 | UGT | 17.80 | 0.06 | 36.01 | 0.15 | pos |
| 2202061 | 7/02/2022 | O/Phx | 21.28 | 0.13 |  |  | neg |
| 2202061 | 7/02/2022 | UGT | 21.18 | 0.05 |  |  | neg |

S5 Table. Isothermal testing of DNA samples.

|  | *LAMP* | | *qPCR* | |  |
| --- | --- | --- | --- | --- | --- |
| *Sample* | *Time (min:sec)* | *Melt (°C)* | *mean Ct* | *Ct S.D.* | *Melt (°C)* |
| K1 OCC | 0 | 0.00 | 0.00 | 0.00 |  |
| K1 UGT | 0 | 0.00 | 0.00 | 0.00 |  |
| K1 REC | 0 | 0.00 | 0.00 | 0.00 |  |
| K2 OCC | 0 | 0.00 | 0.00 | 0.00 |  |
| K2 UGT | 0 | 0.00 | 0.00 | 0.00 |  |
| K2 REC | 27:45 | 87.62 | 34.43 | 0.97 | 81 |
| K3 OCC | 0 | 0.00 | 0.00 | 0.00 |  |
| K3 UGT | 0 | 0.00 | 0.00 | 0.00 |  |
| K3 REC | 0 | 0.00 | 0.00 | 0.00 |  |
| K4 OCC | 17:45 | 87.42 | 0.00 | 0.00 |  |
| K4 UGT | 20:15 | 87.52 | 32.67 | 0.00 | 81 |
| K5 OCC | 0 | 0.00 | 0.00 | 0.00 |  |
| K5 UGT | 20:30 | 87.46 | 27.31 | 0.04 | 81 |
| K5 REC | 0 | 0.00 | 32.96 | 0.14 | 81 |
| K6 OCC | 0 | 0.00 | 0.00 | 0.00 |  |
| K6 UGT | 0 | 0.00 | 0.00 | 0.00 |  |
| K7 OCC | 0 | 0.00 | 0.00 | 0.00 |  |
| K7 UGT | 0 | 0.00 | 0.00 | 0.00 |  |
| K8 OCC | 0 | 0.00 | 0.00 | 0.00 |  |
| K8 UGT | 0 | 0.00 | 34.25 | 0.53 | 81 |
| K9 OCC | 0 | 0.00 | 0.00 | 0.00 |  |
| K9 UGT | 0 | 0.00 | 0.00 | 0.00 |  |
| K9 UGT 2 | 0 | 0.00 | 0.00 | 0.00 |  |
| K10UGT | 0 | 0.00 | 0.00 | 0.00 |  |
| K10 REC | 0 | 0.00 | 0.00 | 0.00 |  |
| K11 OCC | 0 | 0.00 | 0.00 | 0.00 |  |
| K11 UGT | 0 | 0.00 | 0.00 | 0.00 |  |
| K12 OCC | 0 | 0.00 | 0.00 | 0.00 |  |
| K13 UGT | 0 | 0.00 | 0.00 | 0.00 |  |
| K14 OCC | 0 | 0.00 | 0.00 | 0.00 |  |
| K15 OCC | 27:45 | 87.46 | 33.67 | 0.50 | 81 |
| K15 UGT | 0 | 0.00 | 0.00 | 0.00 |  |
| K16 OCC | 0 | 0.00 | 0.00 | 0.00 |  |
| K16 NASAL | 0 | 0.00 | 0.00 | 0.00 |  |
| K17 UGT | 0 | 0.00 | 0.00 | 0.00 |  |
| K18 UGT | 0 | 0.00 | 0.00 | 0.00 |  |
| K19 UGT | 12:00 | 87.58 | 24.65 | 0.10 | 81 |
| K19 LE | 0 | 0.00 | 0.00 | 0.00 |  |
| K20 LE | 0 | 0.00 | 0.00 | 0.00 |  |
| K20 RE | 0 | 0.00 | 0.00 | 0.00 |  |
| K20 UGT | 20:45 | 87.52 | 34.39 | 0.05 | 81 |
| K21 UGT | 0 | 0.00 | 0.00 | 0.00 |  |
| K22 OCC | 0 | 0.00 | 0.00 | 0.00 |  |
| K22 UGT | 0 | 0.00 | 0.00 | 0.00 |  |
| K23 OCC | 0 | 0.00 | 0.00 | 0.00 |  |
| K24 UGT | 0 | 0.00 | 0.00 | 0.00 |  |
| K25 OCC | 0 | 0.00 | 0.00 | 0.00 |  |
| K25 UGT | 15:15 | 87.61 | 23.13 | 0.01 | 80.5 |
| K26 OCC | 0 | 0.00 | 0.00 | 0.00 |  |
| K26 UGT | 0 | 0.00 | 0.00 | 0.00 |  |
| K27 OCC | 22:30 | 87.32 | 33.69 | 0.59 | 81 |
| K27 UGT | 0 | 0.00 | 0.00 | 0.00 |  |
| K28 OCC | 0 | 0.00 | 0.00 | 0.00 |  |
| K28 UGT | 0 | 0.00 | 0.00 | 0.00 |  |
| K29 UGT | 0 | 0.00 | 0.00 | 0.00 |  |
| K30 OCC | 0 | 0.00 | 33.92 | 0.54 | 81 |
| K30 UGT | 0 | 0.00 | 0.00 | 0.00 |  |
| K31 OCC | 27:15 | 87.28 | 32.89 | 0.51 | 81 |
| K31 UGT | 0 | 0.00 | 0.00 | 0.00 |  |
| K32 OCC | 0 | 0.00 | 0.00 | 0.00 |  |
| K32 UGT | 18:45 | 87.52 | 30.15 | 0.10 | 81 |
| K33 EYE | 0 | 0.00 | 0.00 | 0.00 |  |
| K33 UGT | 0 | 0.00 | 0.00 | 0.00 |  |
| K34 UGT | 0 | 0.00 | 0.00 | 0.00 |  |
| K35 UGT | 0 | 0.00 | 0.00 | 0.00 |  |
| K36 UGT | 0 | 0.00 | 0.00 | 0.00 |  |
| K37 EYE | 0 | 0.00 | 0.00 | 0.00 |  |
| K37 UGT | 23:16 | 87.50 | 34.62 | 0.23 | 81 |
| K38 EYE | 0 | 0.00 | 0.00 | 0.00 |  |
| K38 UGT | 0 | 0.00 | 0.00 | 0.00 |  |

S6 Table. Isothermal testing of rapidly processed swabs.

|  | *LAMP* | | *qPCR* | |  | *Spiked LAMP (10^3 copies)* | | *Spiked qPCR (10^3 copies)* | |
| --- | --- | --- | --- | --- | --- | --- | --- | --- | --- |
| *Sample* | *Time (min:sec)* | *Melt (°C)* | *mean Ct* | *Ct S.D.* | *Melt (°C)* | *Time (min:sec)* | *Melt (°C)* | *mean Ct* | *Melt (°C)* |
| K1 LE | 0 | 0 | 0 | 0 |  |  |  |  |  |
| K1 UGT | 0 | 0 | 0 | 0 |  | 15:18 | 87.62 | 27.25 | 81.00 |
| K1 REC | 0 | 0 | 0 | 0 |  | NA | NA | 32.35 | 81.00 |
| K2 RE | 0 | 0 | 0 | 0 |  |  |  |  |  |
| K2 UGT | 0 | 0 | 0 | 0 |  |  |  |  |  |
| K2 REC | 0 | 0 | 0 | 0 |  | NA | NA | NA | NA |
| K3 UGT | 19:03 | 87.34 | 28.36 | 0.273 | 81.00 |  |  |  |  |
| K3 REC | 0 | 0 | 0 | 0 |  |  |  |  |  |
| K4 RE | 0 | 0 | 0 | 0 |  | 14:35 | 87.70 | 28.34 | 81.00 |
| K4 UGT | 0 | 0 | 0 | 0 |  |  |  |  |  |
| K5 E | 0 | 0 | 0 | 0 |  |  |  |  |  |
| K5 UGT | 16:47 | 87.52 | 30.34 | 0.246 | 81.00 |  |  |  |  |
| K6 LE | 0 | 0 | 0 | 0 |  | 16:02 | 87.73 | 28.85 | 81.00 |
| K6 UGT | 0 | 0 | 0 | 0 |  |  |  |  |  |
| K6 REC | 6:43 | 87.67 | 0 | 0 |  |  |  |  |  |
| K7 LE | 0 | 0 | 0 | 0 |  |  |  |  |  |
| K7 RE | 0 | 0 | 0 | 0 |  | 11:28 | 87.52 | 26.52 | 81.00 |
| K7 UGT | 0 | 0 | 0 | 0 |  | 14:46 | 87.68 | 28.64 | 81.00 |
| K8 NASAL | 0 | 0 | 0 | 0 |  |  |  |  |  |
| K8 UGT | 0 | 0 | 0 | 0 |  |  |  |  |  |
| K9 LE | 0 | 0 | 0 | 0 |  |  |  |  |  |
| K9 UGT | 0 | 0 | 0 | 0 |  | 14:46 | 87.65 | 27.02 | 81.00 |
| K10 RE | 0 | 0 | 0 | 0 |  | 15:34 | 87.62 | 27.22 | 81.00 |
| K10 UGT | 0 | 0 | 0 | 0 |  | 17:46 | 87.70 | 27.82 | 81.00 |
| K11 RE | 0 | 0 | 0 | 0 |  |  |  |  |  |
| K11 LE | 0 | 0 | 0 | 0 |  | 15:30 | 87.62 | 28.14 | 81.00 |
| K11 UGT | 0 | 0 | 0 | 0 |  |  |  |  |  |
| K12 UGT | 0 | 0 | 0 | 0 |  | 16:59 | 87.68 | 27.32 | 81.00 |
| K13 LE | 0 | 0 | 0 | 0 |  |  |  |  |  |
| K14 UGT | 0 | 0 | 0 | 0 |  | 21:44 | 87.61 | 29.25 | 81.00 |
| K15 LE | 0 | 0 | 0 | 0 |  |  |  |  |  |
| K15 RE | 0 | 0 | 0 | 0 |  |  |  |  |  |
| K16 E | 0 | 0 | 34.56 | 0.298 | 81.00 |  |  |  |  |
| K16 UGT | 0 | 0 | 0 | 0 |  | NA | NA | 33.54 | 81.00 |
| K17 E | 0 | 0 | 0 | 0 |  |  |  |  |  |
| K17 UGT | 27:15 | 87.78 | 31.89 | 0.746 | 81.00 |  |  |  |  |

NA- no amplification.


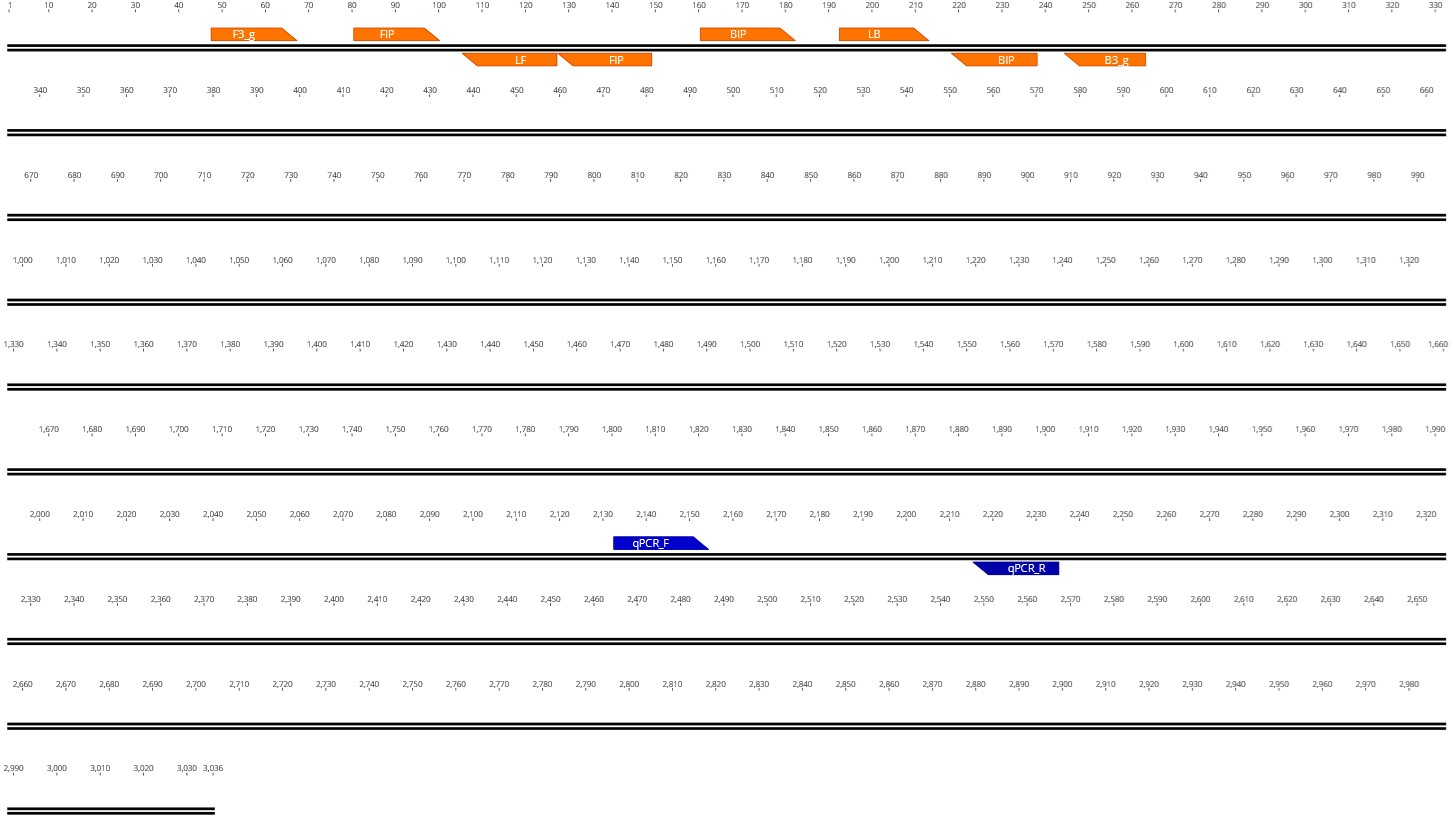
S1 Fig. PhaHV-1 qPCR and LAMP primers positions on the target gene.


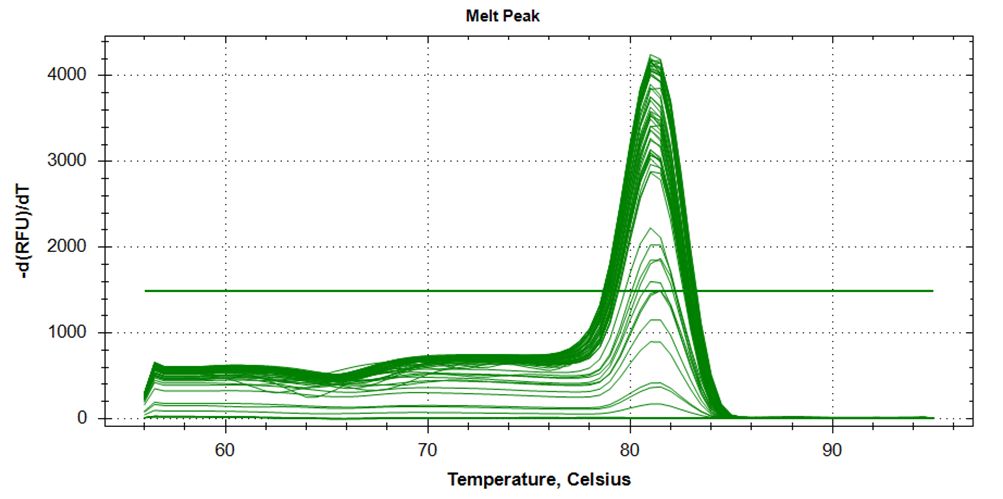


S2a Fig. Melt curves for PhaHV-1 synthetic positive control across serial dilutions and replicates as depicted in Table S1. Efficiency = 97.98%, R^2^ = 0.988, slope = -3.376.





S2b Fig. Limit of detection of PhaHV-1 qPCR assay with PhaHV-1 synthetic positive control copies/reaction plotted on a log scale against average Ct value (Table S3).
